# The relationship between the principals’ emotional intelligence and conflict management: based on latent profile analysis

**DOI:** 10.3389/fpsyg.2025.1548185

**Published:** 2025-04-02

**Authors:** Jinshan Zhou, Sihan Qin, Tingting Jia, Mingxin Shen, Han Liu, Wenlong Tian, Jun Wang

**Affiliations:** ^1^School of Education, Hainan Normal University, Hainan, China; ^2^School of Education, Minzu University of China, Beijing, China

**Keywords:** latent profile analysis, principal, emotional intelligence, conflict management behavior, organizational performance

## Abstract

**Introduction:**

The emotional intelligence (EI) of principals is a critical factor influencing leadership effectiveness and school management. This study aims to explore the heterogeneity of principals’ EI and investigate the differences in conflict management behaviors among principals with varying EI traits.

**Methods:**

A total of 363 principals from 27 provinces and autonomous regions in China were recruited for this study. The Emotional Intelligence Scale (WLEIS) and the Conflict Management Test (M8L4) were used to assess principals’ EI and conflict management behaviors, respectively. Latent profile analysis (LPA) was employed to identify distinct EI profiles among the participants.

**Results:**

The LPA revealed three distinct EI profiles among principals: “low EI,” “middle EI,” and “high EI.” Significant differences were observed in conflict management behaviors across these profiles, particularly in problem-solving, forcing, and avoiding behaviors. Principals with higher EI levels demonstrated more effective problem-solving strategies, while those with lower EI levels tended to rely more on forcing or avoiding behaviors.

**Discussion:**

The findings highlight that the differences in EI among Chinese principals are primarily reflected in their levels, which significantly influence their conflict management approaches. These results underscore the importance of emphasizing EI in the selection and training of school principals. Enhancing EI can promote effective conflict resolution and improve overall school management efficiency.

## 1 Introduction

Overall, the principal, as the leader of the school, undoubtedly plays a very important role in the modern school education system. In China, principals are more often considered to play a critical role in guiding school development, implementing curriculum reform and promoting teacher development (Manhong et al., 2017). And many foreign researches have also found that principals have a significant impact on student achievement, teacher well-being, teaching practice, and organizational health (Liebowitz and Lorna, 2019). In modern society, with the school education ecology becoming increasingly complex, the school is less a closed system composed of teachers, students, principals and other personnel, than an open system closely connected with the social external environment. Individuals with different cultural and social attributes converge in school, the special organizational structure, engaging in interactions as participants in school activities. Therefore, conflicts caused by differences in the roles of the participants are difficult to avoid, both in the coordination process of organizational relations within the school and in the interaction with the outside world. Conflict often leads to wasted time and difficulty in forming organizational consensus, which in turn leads to negative behaviors such as absenteeism, complaints, disagreements, and boycott (Moh Badruddin, 2021). As the main manager of the school, the principal is not only the center of the management of interpersonal conflicts within the school (Moh Badruddin, 2021), but also the bridge to coordinate the relationship between the school and external stakeholders (such as parents, communities, educational policy makers, etc.) In this sense, principals should not only deal with internal conflicts effectively, but also exert conflict management skills in the face of external pressures and multiple demands to reduce the negative impact of conflicts and maintain a harmonious relationship between the school and the outside world (Moh Badruddin, 2021; Tamunodiepiriye et al., 2022). In fact, the type of conflict and the corresponding management behavior have a profound impact on school performance (Saiti, 2014). In order to solve these interpersonal conflicts and even give full play to their possible effects to promote the healthy development of the school, the key undoubtedly lies in the school leaders, especially the principal, who are the main administrators of the school, are bound to assume the responsibility of effectively managing the relevant interpersonal relationships (Winardi et al., 2022).

As for how to effectively conduct conflict management, the key is emotional intelligence (EI). The function of EI is to enable individuals to identify and regulate their emotions through self-monitoring, so as to better cope with work challenges (Mariyadas and Saravanakumar, 2023b). Previous studies have suggested that EI (such as problem-solving ability, social responsibility and impulse control ability) is closely related to the conflict management style of managers (Winardi et al., 2022). And managers with high EI are better able to cope with conflict at work (Sharma, 2019). In addition, many studies have shown that EI is also an important component of principal leadership. Principals with high EI can effectively resolve conflicts and promote organizational harmony through positive communication and relationship maintenance strategies. Principals with low EI are more likely to adopt negative conflict coping styles, which will adversely affect the overall management of the school (Sepiriti, 2023). However, there is still a research gap in studies related to principals’ EI and conflict management. Firstly, the research on the relationship between EI and conflict management behavior of managers (such as principals) in the educational field is relatively limited, and relevant empirical research is particularly scarce. Secondly, the limited relevant research results mostly adopt the variation-centered method, ignoring the heterogeneity within the principals group, which leads to a difficulty in providing targeted suggestions for the professional development of principals from the perspective of the principals group. Therefore, this study uses the individual-centered latent profile analysis method to construct latent profile models based on different dimensions of EI, in order to identify the internal structure, characteristics and differences of EI in school principals, and on this basis, to examine and explore the relationship between EI and conflict management behavior.

## 2 Literature review

### 2.1 Conflict management behavior

Conflict is defined as differences and antagonisms in goals, perceptions, or emotions that occur within individuals or groups, between individuals, between individuals and groups, or between groups and groups (Pondy, 1967; Jehn, 1997; Zarankin, 2008). Because conflict is often difficult to resolve immediately and can lead to a series of negative effects, organizations always try to avoid conflict (Jehn, 1997; Nair, 2008). Pondy (1967) constructed a model of organizational conflict, dividing organizational conflict into three types of conflict: interest negotiation conflict among interest groups, superior and subordinate conflict within bureaucratic organizations, and horizontal systemic conflict among members at the same level. Additionally, he identified five stages of conflict development: latency, perception, feeling, expression, and consequence. Priem and Price (1991) classified conflict into two categories: cognitive-task-related conflict and socio-emotional conflict. Jehn (1995) further defined three types of organizational conflicts: “Relationship conflicts focus on interpersonal relationships, task conflicts focus on the content and goals of work, and process conflicts focus on how to complete tasks”(Jehn, 1997). And his study showed that process and relationship conflicts are negatively correlated with individual satisfaction and team performance, and that task conflict is positively correlated with team performance. Consequently, it could be seen that whether these conflicts are beneficial or harmful depends on the type of conflict, the structure of the group, the interdependence of tasks, and the norms of the group. In addition, previous studies have suggested that task conflict can improve organizational performance (Bourgeois III, 1985; Eisenhardt and Schoonhoven, 1990), decision-making results and team productivity (Amason, 1996), but chronic interpersonal conflict is detrimental to the functioning of an organization, especially in the conflict of negativity, distrust, frustration, and hatred. For example, the disagreement of group members on specific tasks may lead to emotional opposition and exclusion, which can subsequently transfer into the personality problems of individual members (Jehn, 1997). As a result, conflict is not only a disorder of organizational function, but also a problem that needs to be resolved through negotiation or organizational restructuring (Nair, 2008).

In the modern school system, conflict is omnipresent. Alabu et al. (2020) once investigated the conflict management behavior of middle school principals on teachers’ job satisfaction, the study reveals that managers devote a considerable amount of time to managing conflicts (Alabu et al., 2020). However, different from the destructiveness and harmfulness of conflict recognized by the traditional conflict view, the modern conflict view treats conflict with dichotomy, dividing the result of conflict into two kinds: constructive and destructive. Constructive conflict often brings positive results, which could be manifested as people in conflict will improve their perception of fairness and job satisfaction (Behfar et al., 2008), which makes it easier to achieve organizational goals (Udod et al., 2020). However, people in destructive conflicts often feel nervous and anxious (Timothy et al., 2019), which not only affects individual health and happiness, but also reduces work performance and satisfaction (Dimas and Lourenço, 2011). This decline in work performance and satisfaction can subsequently reduce the enthusiasm and performance level of students (Dolgova et al., 2019). After conducting a survey of team performance and management in 170 schools in Israel, Somech et al. (2009) noted that it is not just the existence of conflict, but how people deal with and manage their conflict that greatly influences whether conflict is constructive or destructive. Therefore, understanding the types and sources of conflicts and choosing appropriate conflict management behaviors can effectively resolve conflicts and prevent destructive conflicts (Sepiriti, 2023).

In the context of Chinese culture, “obedience” is often regarded as a core social value due to the influence of the concept of “superior and inferior” in the traditional society (Pan, 2008). This value emphasizes obedience to authority, position in the social hierarchy and the following of superiors (Wu, 2017), which makes Chinese people tend to seek third parties with higher status for mediation in conflict situations (Giebels and Yang, 2009).This strategy of third-party intervention not only helps to relieve the pressure brought by conflict, but also improves individual happiness, so it is considered an effective way of conflict management (Giebels and Janssen, 2020). In education system, the principal’s key role in conflict management as a third-party mediator also has a solid realistic basis (Umigiarni et al., 2021). In other words, in the Chinese education system, due to the influence of the bureaucratic principal responsibility system, the principals often have a great influence in the school. Therefore, the principals can easily play the role of the most important third party in managing various conflicts in the school. But it is evident that he behavior of principals in the face of conflict may vary (Eberts and Stone, 1988). Psychoanalyst Horney (1945), described the basic behavioral tendency of people in the face of conflict. The three tendencies are: close to people, against people, and away from people (Horney, 1945). Accordingly, it could be seen that the intervention behavior of the third-party leader is a combination of problem solving behavior (close to people) and forcing behavior (against people; Conlon et al., 1994). The authoritarian behavior of third-party leaders (against people) means imposing solutions between the conflict parties, the problem solving behavior (close to people) means that principals can understand the concerns of the conflict parties and guide them to the appropriate solution (Römer et al., 2012),and the avoiding behavior (away from people) means that principals may try to avoid getting involved in a conflict when they feel threatened. In this study, we examined the significant differences in three corresponding third party behaviors of leaders in educational Settings at different levels of EI: problem solving, forcing, and avoiding.

### 2.2 Emotional intelligence

The theory of EI is based on the concept of social intelligence proposed by Thorndike in 1920. The models of EI can be divided into three categories: trait model, ability model and mixed model. The trait model, proposed by Petrides, refers to an individual’s self-cognition of his or her emotional capacity, including behavioral tendency and self-perceived ability, measured by self-report (Petrides and Furnham, 2001). Because this model depends largely on self-assessment, it is not widely accepted. The ability model was developed by Mayer and Salovey, who put forward a comprehensive theory of EI and originally defined EI as a subsystem of social intelligence that involves the ability to monitor one’s own and others’ feelings and emotions, distinguish different emotions, and use them to guide one’s thoughts and actions (Salovey and Mayer, 1990). The mixed model, based on the ability model, adds other personality traits and performance theories. This model defines EI as “the capacity for recognizing our own feelings and those of others, for motivating ourselves, and for managing emotions well in ourselves and in our relationships” (Goleman, 1998). In this framework, EI is divided into four domains:

1. Appraisal and expression of emotion in the self;
2. Appraisal and recognition of emotion in others;
3. Regulation of emotion in the self; and
4. Use of emotion to facilitate performance (Wong et al., 2007).

The three models of EI are all used to measure individual emotional ability (Guillén Ramo, 2009). Goleman (1998) pointed out that emotion plays a crucial role in the workplace. He noted that emotions are inherently contagious, capable of disseminating swiftly among individuals, thereby influencing the overall workplace atmosphere (Goleman, 1998). As a result, individuals within the organization use their own feelings about the reactions of others to react in the best direction (Goleman, 1998). In the context of organizational conflict, Bodtker and Katz Jameson (2001) asserted that conflicts among employees should be emotionally stimulated and activated (Bodtker and Katz Jameson, 2001). Therefore, Goleman (1998) regarded social skills as the reason for processing the basic awareness of others’ emotions (Goleman, 1998). Employees with high EI should use this ability to deal with disagreements and provide solutions through open discussion and negotiation (Winardi et al., 2022). Since Goleman’s study was proposed based on the social and emotional competency in the organizational context, the nature of Goleman’s mixed model is more in line with the purpose of this study, so the mixed model of EI was selected for this study. Ali et al. (2022) proposed that emotional intelligence can significantly affect the conflict management of middle school principals, and the impact of emotional intelligence on different conflict management is different (Ali et al., 2022). In a study of 50 principals and 300 teachers, Khattak et al. (2017) found that leaders have a strong sense of emotional intelligence and are able to manage the disruptive emotions of their subordinates. Besides, they can utilize the abilities of teachers in the most effective way (Khattak et al., 2017). Therefore, in the face of conflict, principals with different degrees of EI have different conflict management behaviors, which in turn will affect the effectiveness of conflict management behaviors.

Compared with previous studies, this study goes beyond the individual or group perspective of previous studies (Jordan and Troth, 2021), and actively considers the important role of emotional intelligence of third-party leaders on conflict management behavior from the perspective of third-party leaders. Moreover, compared with the previous variance-centered methods, this study adopts latent profile analysis, an individual-centered method that focuses more on the heterogeneity of the subjects, to better identify the differences within the group of principals and the obvious different characteristics and reaction patterns of principals with different EI levels in conflict management behaviors. Thus, the following hypotheses are proposed.

*H*1. There are significant differences in the shape (1a) and level (1b) of the latent profile model of EI of principals in our country, and principals with different levels of EI have different latent characteristics.

*H*2. There is a positive correlation between Forcing behavior (2a), Avoiding behavior (2b) and Problem solving behavior (2c) of third-party conflict management behaviors and four dimensions of EI: Appraisal and expression of emotion in the self; Appraisal and recognition of emotion in others; Regulation of emotion in the self; Use of emotion to facilitate performance.

*H*3. There are significant differences in conflict management behaviors among principals with different EI levels.

## 3 Methods

### 3.1 Participants and procedures

The samples in this study were from 27 provinces and autonomous regions in China. After approval, the researchers introduced the project online and recruited principals who volunteered to participate in this survey. Data collection took place between June 23 and July 20, 2024. Once principals agreed to participate in this program, they were asked to fill out the WLEIS and M8L4 questionnaires and submit them online. A total of 540 questionnaires were distributed in this study, 363 of which were valid questionnaires and 177 were invalid questionnaires, with a questionnaire recovery rate of 67.2%. Descriptive statistics of the sample are presented in Table 1.

**Table 1:** Descriptive statistics of the sample (*N* = 363).

Items	Categories	N	%
Gender	Male	262	72.2
	Female	101	27.8
School Level	Primary School	221	60.9
	Middle School	101	27.8
	High School	41	11.3
School locality	Village	71	19.6
	Town	125	34.4
	Country Town	124	34.2
	Large City	43	11.8
Type of School	Public School	345	95.0
	Private School	18	5.0
Years of Service as Principal	0–5 years	201	55.4
	6–10 years	78	21.5
	11–15 years	39	10.7
	16–20 years	26	7.2
	21 years and above	19	5.2

### 3.2 Instruments

The two instruments used in this study are the Chinese versions of Wong and Law’s Emotional Intelligence Scale (WLEIS) and the Conflict Management Test (M8L4) developed by East Carolina University in the United States, both of which are used in the form of teachers. The WLEIS and M8L4 were translated into Mandarin following a standardized forward-backward procedure (Brislin, 1970), with all items culturally adapted to align with the educational leadership context in mainland China (e.g., replacing “colleagues” with “teaching staff”).

WLEIS consists of 16 items, which are divided into four EI domains: Appraisal and expression of emotion in the self; Appraisal and recognition of emotion in others; Regulation of emotion in the self; Use of emotion to facilitate performance. Each domain contains four items. Responses are made on a 5-point Likert scale, with “1” indicating “strongly disagree” and “5” indicating “strongly agree.” The reliability and validity of the WLEIS scale have been tested in a large number of studies in Hong Kong and the mainland (Chi-Sum and Wong, 2010; Yin et al., 2013). The scale has a Cronbach’s alpha of 0.930. The Cronbach’s alpha for appraisal and expression of emotion in the self is 0.887, regulation of emotion in the self is 0.859, use of emotion to facilitate performance is 0.836, and for appraisal and recognition of emotion in others is 0.922. All of these values are greater than 0.8, suggesting that the reliability of the scale is excellent (Cronbach, 1951; Nunnally and Bernstein, 1994). The construct validity of the scale is also highly satisfactory (χ^2^ = 227.516, df = 98, χ^2^/df = 2.322, CFI = 0.966, TLI = 0.959, RMSEA = 0.060).

The Conflict Management Test (M8L4) consists of 15 questions, using a 5-point Likert scale. In its original form, the Cronbach’s alpha of the scale ranged from 0.39 to 0.70. In this study, a confirmatory factor analysis (CFA) was performed to compare the two models. The Cronbach’s alpha of the adjusted three-factor model was 0.615, which was greater than 0.6, indicating that it was within the acceptable range (Jehn, 1995; Long and Zhou, 2004a; Jung and Wickrama, 2008; Ma et al., 2008; Ann and Yang, 2012; Asparouhov and Muthén, 2021). The data showed that the adjusted three-factor model of forcing behavior, avoiding behavior and problem solving behavior was more suitable for this study than the original five-factor model, because the construct validity of the original model was χ^2^ = 347.093, df = 80, χ^2^/df = 4.339, CFI = 0.777, TLI = 0.707, RMSEA = 0.096 and that of the adjusted model was χ^2^ = 33.384, df = 22, χ^2^/df = 1.517, CFI = 0.974, TLI = 0.958, RMSEA = 0.038.

### 3.3 Data analysis

SPSS27.0 was used for data collation, and Mplus8.0 was used for latent profile analysis of emotional intelligence. Starting from 2 categories, the number of model categories was gradually increased until the best fitting model was found. The model fitting test indexes include AIC, BIC, sample corrected BIC (αBIC), Entropy, LMRT, and BLRT based on Bootstrap. Firstly, the smaller the three information evaluation indexes (AIC, BIC, αBIC), the better the model fit (Nylund et al., 2007). Second, *p*-values for LMR and BLRT reaching significance levels indicate that the K-category model is superior to the k-1 category model (Jung and Wickrama, 2008). Furthermore, Entropy closer to 1 indicates a more accurate classification. Entropy equal to 0.8 indicates that the classification accuracy is more than 90%, with high accuracy. It should be noted that the minimum category proportion is not less than 5% of the total sample or the number of people is not less than 30. After completing the latent profile analysis of EI, the outcome variable of “conflict management behavior” was included, and the relationship between this variable and the latent category of EI was analyzed by BCH method in Mplus. The BCH method in the Mplus program is actually the modified BCH method (Asparouhov and Muthén, 2021). Then, on this basis, the BCH method was used to explore the differences in conflict management behaviors of principals under different EI categories by variance analysis of the chi-square test.

## 4 Results

### 4.1 Common method bias

This study used self-reporting to collect data. Although the test process was strictly controlled, common method bias still needed to be tested. Therefore, Harman single factor method was used (Long and Zhou, 2004b). Unrotated exploratory factor analysis was performed on all measures of EI and conflict management behavior, and six common factors with a characteristic root greater than 1 were extracted. The first principal component explained 33.79% of the total variance variation, which was lower than the critical value of 40%. Therefore, it is inferred that common method bias does not seriously affect the conclusions of this study.

### 4.2 Descriptive statistics and correlation analysis

Descriptive statistics and bivariate intercorrelations among study variables are shown in Table 2. The correlation coefficients between forcing behavior and the four dimensions of EI are as follows: 0.22, 0.29, 0.34, and 0.20, all of which are significantly positive correlations (*p* < 0.01), showing that hypothesis 2a is supported. The correlation coefficients between avoiding behavior and the appraisal and expression of emotion in the self, as well as the regulation of emotion in the self, are 0.12 and 0.12, respectively, and are significantly positive correlations (*p* < 0.05). The correlation coefficient between avoiding behavior and use of emotion to facilitate performance are 0.04, showing a positive correlation. The correlation coefficient between avoiding behavior and use of emotion to facilitate performance is −0.02, indicating a negative correlation. Therefore, hypothesis 2b is not supported. The correlation values between problem solving behavior and appraisal and expression of emotion in the self, regulation of emotion in the self, and use of emotion to facilitate performance are 0.34, 0.32, and 0.28, respectively, showing a significant positive correlation (*p* < 0.01). The correlation coefficient between problem solving behavior and the appraisal and recognition of emotion in others is 0.13, and is significantly positive (*p* < 0.05). Therefore, hypothesis 2c is supported.

**Table 2:** Descriptive statistics and bivariate intercorrelations among study variables.

Dimension	1	2	3	4	5	6	7
1. Appraisal and expression of emotion in the self	1.00						
2. Regulation of emotion in the self	0.63 ^**^	1.00					
3. Use of emotion to facilitate performance	0.60 ^**^	0.53 ^**^	1.00				
4. Appraisal and recognition of emotion in others	0.51 ^**^	0.49 ^**^	0.56 ^**^	1.00			
5. Forcing	0.22 ^**^	0.29 ^**^	0.34 ^**^	0.20 ^**^	1.00		
6. Avoiding	0.12 ^*^	0.12 ^*^	0.04	−0.02	0.08	1.00	
7. Problem solving	0.34 ^**^	0.32 ^**^	0.28 ^**^	0.13 ^*^	0.16 ^**^	0.44 ^**^	1.00
M	4.16	3.92	4.09	3.93	2.49	2.31	2.87
SD	0.71	0.68	0.65	0.73	0.56	0.66	0.62

*N* = 363. *p < 0.05, **p < 0.01.

### 4.3 Latent profile analysis of emotional intelligence

A potential profile model was established with the four dimensions of EI, appraisal and expression of emotion in the self, regulation of emotion in the self, use of emotion to facilitate performance, appraisal and recognition of emotion in others, as the manifest variables. Since the BCH method does not support 1-category, 2–6 categories were set for latent profile model fit estimation. After comprehensive consideration, 3-category is selected as the optimal model for the following reasons: (a) AIC, BIC, and ABIC values decreased as the number of profiles increased, and the 3-category solution reached the point where the overall model fit rate began to decline (Table 3); (b) The LMRT values indicated that the solutions for 4 categories or more were not superior to the 3-category solution in quality; (c) The Entropy value of the 3-category solution was the highest, indicating that the 3-category solution was superior to the others; and (d) The average probability of participants being assigned to the 3 potential categories was all above 0.96, while the probability of being assigned to other groups was all below 0.05 (see Table 4), further confirming the accuracy of the 3-category model.

**Table 3:** Comparison of models for latent profiles (*N* = 363).

Profile number	AIC	BIC	ABIC	Entropy	LMR	BLRT	Sample size per profile
2	2,656.235	2,706.863	2,665.619	0.768	0.0000	0.0000	202;161
**3**	**2,444.304**	**2,514.403**	**2,457.297**	**0.947**	**0.0000**	**0.0000**	**67;158;138**
4	2,408.296	2,497.867	2,424.899	0.883	0.1947	0.0000	155;67;95;46
5	2,363.985	2,473.029	2,384.197	0.878	0.1414	0.0000	41;39;142;48;93
6	2,346.259	2,474.774	2,370.080	0.879	0.1391	0.0000	39;144;40;51;10;79

The bolded entries represent the fit statistics for the selected solution in the current study. AIC, Akaike information criterion; BIC, Bayesian information criterion; ABIC, sample size-adjusted BIC; BLRT, bootstrap 302 likelihood ratio test; LMRT, Lo-Mendell-Rubin likelihood ratio test.

**Table 4:** Latent profiles and classification accuracy of students in each profile.

Profiles	n	%	Probability		
			Class 1	Class 2	Class 3
Class 1	67	18.50	0.963	0.037	0.000
Class 2	158	43.79	0.017	0.976	0.008
Class 3	138	37.70	0.000	0.017	0.983

Class 1 = low EI, Class 2 = middle EI, Class 3 = high EI, the same as follows.

**Figure 1:**
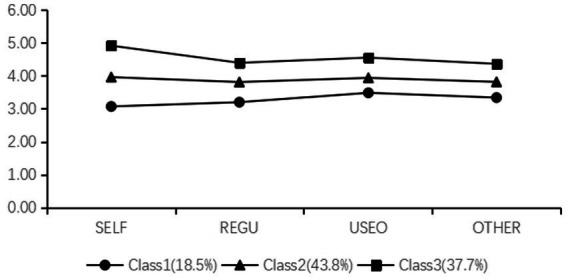
Illustration of the latent profiles in the selected 3-profile solution based on standardized scores.

In addition, the distribution of potential categories of appraisal and expression of emotion in the self, regulation of emotion in the self, use of emotion to facilitate performance, and appraisal and recognition of emotion in others, is shown in Figure 1. According to the score characteristics of each category in the four dimensions, the 3-category was potentially named. The first profile was characterized by the lowest levels of appraisal and expression of emotion in the self, regulation of emotion in the self, use of emotion to facilitate performance, and appraisal and recognition of emotion in others, so this kind was labeled as “low EI”(*n* = 67, 18.5%). The second profile was characterized by a moderate levels of the four dimensions of EI. As such, this profile was labeled as “middle EI” (*n* = 158, 43.79%). The third profile was characterized by the highest levels of the four dimensions of EI, so this profile was labeled as “high EI” (*n* = 138, 37.70%). Thus, Hypothesis 1b is supported.

### 4.4 Effects of different latent types of emotional intelligence on conflict management behavior

In order to explore the relationship between the latent categories of the four dimensions of principal’s emotional intelligence and the three types of conflict management behaviors, Mplus was used as the regression mixed model with outcome variables, and the modified BCH results of the overall chi-square test were shown, as well as the chi-square statistics for pairwise comparisons between classes (*N* = 363). According to the overall chi-square test, there are significant differences among the three profiles. The Class 1 group has the lowest score in the low EI level, while the Class 3 group has the highest score in the high EI level. As shown in Table 5, most comparisons between the three groups are significant, but there are a few exceptions: (1) Class 2 and Class 3 have no difference in forcing behavior; (2) Class 1 and Class 2, as well as Class 2 and Class 3, have no difference in avoiding behavior. Therefore, Hypothesis 3 is supported.

**Table 5:** Relations of the three latent profiles to the outcome variables in the full sample (*N* = 363).

Variable^a^	Class 1 (*n* = 67)	Class 2 (*n* = 158)	Class 3 (*n* = 138)	χ^2^	Class 1 vs. Class 2	Class 1 vs. Class 3	Class 2 vs. Class 3
Forcing	2.20 (0.06)	2.50 (0.04)	2.61 (0.05)	26.64^**^	15.15^**^	26.06^**^	2.33
Avoiding	2.14 (0.08)	2.29 (0.05)	2.41 (0.06)	7.26^*^	2.53	7.23^**^	2.03
Problem solving	2.51 (0.07)	2.83 (0.04)	3.11 (0.06)	44.44^**^	13.76^**^	43.24^**^	15.61^**^

Analyzes were performed with BCH and DCAT procedures in Mplus 8. Class 1 = low EI, Class 2 = middle EI, Class 3 = high EI. *p < 0.05, **p < 0.01(two-tailed).

a Relations of the three latent profiles to categorical outcomes variable (i.e., competing, avoiding and accommodating) are presented as probability and standard error (SE). Relations of the three latent profiles to continuous outcomes variables are presented as M (SE).

## 5 Discussion

This study was based on an “individual centered” latent profile analysis (LPA) approach to investigate the levels of different types of EI in school principals and their association with conflict management behaviors. This study is unique in the following ways.

Firstly, based on the “individual-centered” perspective, this study employed the LPA method which focuses on the heterogeneity of the principal group. While various methodologies have been employed to assess EI, the majority of existing research has predominantly utilized quantitative techniques, including multiple regression (20.7%), regression analysis (20.7%), structural equation modeling (17.2%), hierarchical regression (17.2%), correlation (10.3%), and partial least squares (3.4%). Notably, there has been a lack of studies that adopt an “individual-centered” perspective to investigate the group heterogeneity of principals (Winardi et al., 2022). Therefore, this study conducts a latent profile analysis of the four dimensions of principals’ emotional intelligence, taking into account the corresponding indicators and ultimately identifying three latent categories: “low EI,” “middle EI” and “high EI.” It is found that principals with different EI levels have different potential characteristics. The distributional profile of principals’ emotional intelligence reveals distinct patterns: those with low EI (18.5%) exhibit the lowest scores across all four assessment dimensions, while principals with high EI (37.7%) demonstrate superior performance in these dimensions. Principals falling within the middle level of the EI spectrum (43.8 percent) achieve scores that lie between and significantly above average levels. In total, 81.5 percent of principals possess medium or high levels of emotional intelligence, which shows that most principals can handle conflicts well because their EI levels are relatively high.

Secondly, the study examines the relationship between principals’ emotional intelligence and conflict management behavior, further enriching relevant researches. While an increasing number of studies have focused on the relationship between EI and job performance (Ann and Yang, 2012), there has been limited research on the relationship between EI and conflict management behavior (Winardi et al., 2022). Early organizational conflict theorists focused on the causes and resolution of conflicts and discovered the drawbacks of conflicts to organizational operations (Jehn, 1995). Between 1997 and 2006, research primarily concentrated on workplace conflicts, conflict management styles, cultural differences, group conflicts, and job performance (Ma et al., 2008). Between 2007 and 2017, research primarily involved topics such as gender, power in emotions and negotiation, culture and conflict management styles, trust and cooperation, mediation and social conflicts, and performance and management (Caputo et al., 2019). In a literature review on EI and conflict management published in 2021 by Michael Aswin Winardi, Catherine Prentic, and Scott Weaven, the authors paid more attention to geographical location and research area, methods of measuring EI, types of conflicts, and the content of EI in resolving conflicts, and mentioned that limited research has discussed the relationship between EI and conflict management (Winardi et al., 2022). In this study, EI is divided into four dimensions, and based on this, three third-party leader conflict management behaviors are further analyzed. The conclusions are as follows: (1) The four dimensions of EI are significantly positively correlated with problem solving behavior, indicating that the higher the principals’ emotional intelligence, the more likely they are to engage in mediation behavior, which is consistent with previous research findings (Chan et al., 2014; Assi and Eshah, 2023). (2) Appraisal and expression of emotion in the self, regulation of emotion in the self, use of emotion to facilitate performance, are significantly positively correlated with avoiding behavior, while appraisal and recognition of emotion in others is negatively correlated with avoiding behavior, indicating that the higher the principals’ ability to identify and evaluate others’ emotions, the more likely they are to engage in avoiding behavior, which is also consistent with previous research findings (Chan et al., 2014). (3) The four dimensions of EI are significantly positively correlated with forcing behavior, indicating that the higher the EI of principals, the more likely they are to engage in forcing behavior. Although this conclusion has been verified in adolescent populations in recent years (Jintu et al., 2023), it has not been verified in principals (García-Sancho et al., 2014). Therefore, this study is the first to verify this conclusion in principals, and future research can build on this foundation for further investigation.

Thirdly, the principals’ emotional intelligence is studied as a predictor of conflict management behavior, expanding the scope of educational research. Although previous research did not fully explore the role of EI in conflict management behavior (Winardi et al., 2022), conflict management scholars have already foreseen that EI may become a key issue in the field (Caputo et al., 2019). Furthermore, although there has been extensive research on EI and conflict management behavior in educational settings, the behavior of principals as third-party leaders in conflict management has often been ignored. This research fills the existing gap by regarding the principals’ emotional intelligence as a predictive variable. It elucidates the variations in conflict management behaviors exhibited by principals with differing levels of EI, thereby offering novel theoretical insights into conflict management practices within the educational context. The results show that principals with low EI level have the lowest score, while principals with high EI level have the highest score. The three groups show relatively significant differences in different behaviors, with a few exceptions: Class 2 and Class 3 have no difference in forcing behavior; Class 1 and Class 2, Class 2 and Class 3 have no difference in avoiding behavior. Therefore, it could be seen that there is no difference in forcing behavior between principals with middle and high EI level, no difference in avoiding behavior among principals with low, middle and high EI level, and the greatest differences in problem solving behavior among the three groups, which provides references for principals to choose appropriate conflict management behavior.

## 6 Conclusion

This study employed latent profile analysis (LPA) to investigate the heterogeneity of principals’ emotional intelligence (EI) and its association with conflict management behaviors in the Chinese educational context. Firstly, consistent with previous studies (Chan et al., 2014; Assi and Eshah, 2023), this study provides new evidence that principals with high EI level are more likely to engage in problem solving behavior instead of avoiding behaviors. Secondly, what is most noteworthy is that principals with high EI level are more likely to engage in forcing behavior. And in this sense, there is a significant difference among principals with low, middle and high EI level. This study is the first to verify this conclusion in principals, and future research can build on this foundation for further investigation. Furthermore, this study categorizes principals’ emotional intelligence based on principals’ appraisal and expression of emotion in the self, appraisal and recognition of emotion in others, regulation of emotion in the self and use of emotion to facilitate performance, and further examines the differences in conflict management behaviors of principals with different levels of emotional intelligence, which may contribute to a deeper understanding of the relationship between emotional intelligence and the conflict management behaviors of leaders in third-party.

## 7 Practical implications

The present study holds significant implications for advancing the theoretical framework of emotional intelligence and exploring the determinants influencing principals’ conflict management behavior. Drawing upon the findings, several educational recommendations are proposed.

Firstly, it is necessary to enhance the understanding of the impact of principals’ emotional intelligence on conflict management behaviors. As the leaders of the school, principals are responsible for creating passion and motivating followers to perform at their best (Dare and Saleem, 2022), so their EI levels have a great impact on the overall management of the school. In fact, recent studies have affirmed the importance of emotional intelligence (Ali et al., 2022; Mariyadas and Saravanakumar, 2023a), but most studies have not classified the emotional intelligence of principals and explored the different conflict management behaviors of principals with different emotional intelligence. Based on previous studies, this study finds that principals with high EI level are more likely to engage in problem solving behavior instead of avoiding behavior when facing conflicts by classifying the emotional intelligence of principals. Consequently, it is imperative to give greater consideration to the influence of emotional intelligence on conflict management behavior, enabling school principals to effectively leverage emotional intelligence in the resolution of conflicts.

Secondly, it is important to enhance the principals’ ability to select appropriate conflict management behaviors using emotional intelligence. Emotional intelligence is critical to effective leadership, helping leaders be more transformative, make better decisions, effectively manage stress, and create cohesive team dynamics (Gómez-Leal et al., 2021; Koutsioumpa, 2023). This study finds that principals’ behaviors vary with their EI level. There is no difference in compulsive behavior between principals with middle EI level and principals with high EI level, no difference in avoiding behavior among principals with high, middle and low EI level, and the greatest difference in mediation behavior among the three groups. In addition, this study also finds that appraisal and expression of emotion in the self, appraisal and recognition of emotion in others, regulation of emotion in the self, and use of emotion to facilitate performance have a significant effect on the change of the behaviors of the third-party in conflict management. Therefore, it is important for principals to improve their ability in appraisal and expression of emotion in the self, appraisal and recognition of emotion in others, regulation of emotion in the self, and use of emotion to facilitate performance and conduct appropriate conflict management behaviors when facing conflicts.

## 8 Limitations and future directions

Several limitations of this study should be considered. Firstly, this study mainly conducted a cross-sectional study instead of longitudinal research, collecting data at a single time point instead of at multiple time points, so it may restricts the ability to establish causal relationships between principals’ emotional intelligence (EI) and conflict management behaviors. Therefore, in subsequent studies, it will be better to examine the relationship between principals’ emotional intelligence and conflict management behavior by adopting the longitudinal method. In other words, researchers can conduct another survey after a certain period of time and compare the results of the two surveys to verify the relevant relationships. Secondly, in the empirical study of this paper, most variables were evaluated by self-assessment. Although the scales used in this study are all mature scales that have been extensively tested, the adoption of self-report may result in common method variance. Therefore, in future studies, multiple sources of data collection can be considered, and appropriate methods other than self-assessment can be used to measure some variables to increase the reliability of the research results. Third, the study’s exclusive focus on principals within the Chinese cultural context may limit the generalizability of the findings. China’s collectivist cultural norms may uniquely shape principals’ perceptions of emotional intelligence (EI) and their conflict management behaviors, which could differ significantly in individualistic or other distinct cultural settings. Therefore, future research should extend this investigation to diverse cultural contexts to examine whether the identified relationships between EI profiles and conflict management behaviors are universal or culturally contingent.

## Data Availability

The raw data supporting the conclusions of this article will be made available by the authors, without undue reservation.
