# Supplementary material for: The relationship between the principals’ emotional intelligence and conflict management: based on latent profile analysis
Source: Front Psychol. 2025 Apr 2;16:1548185. doi: 10.3389/fpsyg.2025.1548185 (PMC12001525; doi:10.3389/fpsyg.2025.1548185)
Supplement: Supplementary file 1 [file Data_Sheet_1.ZIP › data/Instruments.docx]

**校长中文版问卷**

1. 您的年龄是
2. 您的性别是
3. 您所任职的学校属于哪个学段
4. 您的学校所在区域是
5. 作为校长工作了多少年
6. 情商EI（emotional intelligence）

1. I have a good sense of why I feel certain feelings most of the time.

2. I have a good understanding of my own emotions.

3. I really understand what I feel.

4. I always know whether I am happy or not.

5. I always know my friends' emotions from their behaviour.

6. I am a good observer of others' emotions.

7. I am sensitive to the feelings and emotions of others.

8. I have a good understanding of the emotions of people around me.

9. I always set goals for myself and then try my best to achieve them.

10. I always tell myself I am a competent person.

11. I am a self-motivating person.

12. I would always encourage myself to try my best.

13. I am able to control my temper so that I can handle difficulties rationally.

14. I am quite capable of controlling my own emotions.

15. I can always calm down quickly when I am very angry.

16. I have good control of my emotions.

1. 在大多数时候，我都很清楚自己的感受因何而起。

2. 我很理解自己的情绪。

3. 我真正理解自己的感受。

4. 我总是知道自己是否快乐。

5. 我总能从朋友的行为中了解他们的情绪。

6. 我善于观察他人的情绪。

7. 我对他人的感受和情绪很敏锐。

8. 我能很好地理解周围人的情绪。

9. 我总是为自己设定目标，然后尽最大努力去实现它们。

10. 我总是告诉自己，我是一个有能力的人。

11. 我是一个有自驱力的人。

12. 我总是鼓励自己尽力而为。

13. 我能够控制自己的脾气，从而理性地处理问题。

14. 我有能力控制自己的情绪。

15. 当我非常生气时，我总是能够很快冷静下来。

16. 我对自己的情绪控制的很好。

二、冲突管理CM（conflict management）

1. I explore issues with others to find solutions that meet everyone's needs.

2. I try to negotiate and adopt a "give-and-take"approach to problem situations.

3. I try to meet the expectations of others.

4. I generally argue my case and insist on the merits of my point of view.

5. When there is a disagreement, I gather as much information as I can to keep the lines of communication open.

6. When I find myself in an argument, I usually say very little and try to leave as soon as possible.

7. I try to see conflicts from both sides. What do I need? What does the other person need? What are the issues involved? 8. I prefer to compromise when solving problems and just move on.

9. I find conflicts challenging and exhilarating. I enjoy the battle of wits that usually follows.

10. Being at odds with other people makes me feel uncomfortable and anxious.

11. I try to accommodate the wishes of my friends and family.

12. I can figure out what needs to be done and I am usually right.

13. To break deadlocks, I would meet people halfway.

14. I may not get what I want, but it is a small price to pay for keeping the peace.

15. I avoid hard feelings by keeping my disagreements with others to myself.

1. 与他人一起探讨问题时，我会尽可能选择满足每个人需求的方案。

2. 我总是通过协商并采取“相互妥协”的方式来解决问题。

3. 我总是努力满足他人的期望。

4. 我常常会据理力争，坚持自己观点的优点（价值）。

5. 当出现分歧时，我会尽可能多地收集信息，保持沟通渠道畅通。

6. 当我陷于争论中时，我通常很少说话，并试图尽快离开。

7. 我尝试从双方的角度出发审视冲突。我需要什么？对方需要什么？涉及哪些问题？

8. 在解决问题时，我更倾向于妥协，然后继续推进。

9. 我发现冲突既具有挑战性又令人振奋。我享受在冲突中斗智斗勇。

10. 与他人不和让我感到不舒服和焦虑。

11. 我尽量满足同事和学校其他工作人员的愿望。

12. 我能想出（断定）需要做什么，而且我通常是对的。

13. 为了打破僵局，我愿意与人互相妥协。。

14. 我觉得牺牲自己的需求去维持和平是很小的代价。

15. 我不与他人发生争执，以免引起不快。

三、工作满意度JS Job satisfaction

1. I feel fairly well satisfied with my present job
2. Most days I am enthusiastic about my work
3. Each day of work seems like it will never end. (reverse scored)
4. I find real enjoyment in my work
5. I consider my job rather unpleasant. (reverse scored)

1)我对目前的工作感到相当满意

2)大多数时候我对工作充满热情

3)每天的工作好像没完没了。(反向计分）

4)我在工作中找到了真正的乐趣

5)我觉得我的工作相当不愉快。(反向计分）

四、校长离职意向IQ Intention to quit of school principals

(1)"I think about leaving the organization."

(2)"I am actively looking for other jobs."

(3)"I intend to leave the organization within the next year."

(4)"I am considering quitting my job."

(5)"I do not see myself staying with this organization much longer."

(6)"I would leave this job if I had another offer."

(1) 我想过离开这个学校。

(2) 我正在积极寻找其他工作机会。

(3) 我打算在未来一年内离职。

(4) 我正在考虑辞职。

(5) 我认为自己不会在这个学校待太久。

(6) 如果有其他工作机会，我会离职。

1. conflict-stress relationship CS
2. How often do you feel nervous during or directly after a conflict with colleagues?
3. How often do you become upset during or directly after a conflict with your colleagues?
4. How often does the stress in a conflict with colleagues increase to such high levels that you cannot let go of it?
5. How often do you feel tension during or directly after a conflict with colleagues?

1.在同事发生冲突时或冲突后，您通常会感到不安吗？

2.在与同事发生冲突时或冲突后，您通常会感到心烦意乱吗？

3.在与同事发生冲突时，您通常会感到压力增大，以至于无法释怀吗？

4.在与同事发生冲突时或冲突后，您通常会感到紧张吗？
